# In-Depth on the Fouling and Antifouling of Ion-Exchange Membranes

**DOI:** 10.3390/membranes11120962

**Published:** 2021-12-07

**Authors:** Lasâad Dammak, Natalia Pismenskaya

**Affiliations:** 1 Institut de Chimie et des Matériaux Paris-Est (ICMPE), Université Paris-Est Créteil, CNRS, ICMPE, UMR 7182, 2 Rue Henri Dunant, 94320 Thiais, France; 2 Department of Physical Chemistry, Kuban State University, 149 Stavropol’skaya Str., 350040 Krasnodar, Russia; n_pismen@mail.ru

This work is a synthesis of several in-depth studies on fouling and antifouling phenomena of ion-exchange membranes (IEMs). It is motivated by the increasing interest of the scientific and industrial communities in the diagnosis, quantification, and understanding of the different fouling aspects, and also in applications for its reduction. The main aim of such a synthesis is to help users of processes associated with ion-exchange membranes to find the best solution in order to extend the duration of membrane life and to reduce the operating costs of industrial processes.

Separation processes associated with ion-exchange membranes (IEM-processes), and with their two main families of dialysis (absence of electric current) and electrodialysis (application of an electric current) membranes, were first established for water treatment and desalination, and are still highly recommended in this field for their high water recovery, long life, and acceptable electricity consumption. Today, thanks to technological progress, these IEM-processes, especially electrodialysis, and the emerging new IEMs, have been extended to many other applications (chemical, food, pharmaceutical, and energy industries, etc.). This expansion of uses has also generated several problems, such as limitation of IEMs’ lifetimes due to different ageing phenomena (because of organic and/or mineral compounds). If these aspects are not sufficiently controlled and mastered, the use and the efficiency of IEM-processes will be limited since they will no longer be competitive or profitable compared to other separation methods. The current commercial IEMs have excellent performances in IEM-processes; however, organic foulants such as proteins, surfactants, polyphenols, or other natural organic matter, can adhere to their surface (especially when using anion-exchange membranes (AEMs)) forming a colloid layer or they can infiltrate the membrane matrix, which leads to an increase in ion-transport resistance, resulting in higher energy consumption, lower water recovery, loss of membrane permselectivity and current efficiency as well as lifetime limitation. It has therefore become necessary and very useful to develop research areas to understand these fouling phenomena in order to act appropriately to limit their negative effects. Significant developments have been achieved in recent years and, currently, research is very active on this topic.

In this Special Issue, we have collected contributions on recent advancements in techniques for diagnosis and characterization of the fouling effects on ion-exchange membranes, the mechanisms governing this complex phenomenon, and the various innovative and economically viable solutions for reducing fouling. The Special Issue contains nine articles. Two reviews on ion-exchange membrane fouling during the electrodialysis process in the food industry are included; three studies are focused on the formation of fouling both on the surface and inside the IEMs, one of which involves a mathematical modeling of this phenomenon; three other studies are devoted to the antifouling processes, and the ninth study is dedicated to scaling (mineral fouling).

Dammak et al. [1] and Pismenskaya et al. [2] reviewed a significant number (about 380) of recent and relevant high-quality scientific papers, research articles and reviews studying the phenomena of IEM fouling during the ED process in the food industry with a special focus on the last decade. They first classified the different types of fouling according to the most commonly used classifications. Then, the fouling effects and the characterization methods and techniques as well as the different fouling mechanisms and interactions where presented, analyzed, discussed, and illustrated in a very in-depth manner. The relationships between the nature of the foulant and the structural, physicochemical, and transport properties and behaviors of ion-exchange membranes in an electric field were analyzed using experimental data and modern mathematical models. The implications of traditional chemical cleaning are taken into account in this analysis and modern "mild" membrane cleaning methods are discussed. Therefore, conclusions and outlook are provided, highlighting the main technical challenges, the current status of the fouling and antifouling phenomena of ion-exchange membranes in the food industry, and the key points for the future R&D. Finally, many challenges for the near future are identified and realistic suggestions are made.

Kozmai et al. [3] show the broad possibilities of electrochemical impedance spectroscopy for assessing the capacitance of interphase boundaries and the resistance and thickness of the foulant layer through the example of an AMX-Sb homogeneous membrane separating a red-wine solution from a 0.02 M sodium chloride solution. This enabled them to determine to what extent foulants affect the electrical resistance of ion-exchange membranes, the ohmic resistance and the thickness of diffusion layers, the intensity of water splitting, and the electroconvection in under- and over-limiting current modes. The authors established that short-term (10 h) contact of the AMX-Sb membrane with wine reduces the water splitting due to the screening of fixed groups on the membrane surface by wine components. On the contrary, biofouling, which develops upon a longer membrane operation, enhances water splitting, due to the formation of a bipolar structure on the AMX-Sb surface. This bipolar structure is composed of a positively charged surface of the anion-exchange membrane and negatively charged outer membranes of microorganisms. The island-like distribution of foulants on the membrane surface contributes to the development of electroconvection. Using optical microscopy and microbiological analysis, Kozmai et al. found that more intense biofouling was observed on the AMX-Sb surface that had not been in contact with wine.

Todeschini et al. [4] were interested in the herring milt hydrolysate (HMH) which, like many fish products, has the drawback of being associated with off-flavors. As odor is an important criterion, an effective deodorization method targeting the volatile compounds responsible for off-flavors was developed. First, the authors identified 15 volatile compounds known for their main contribution to the odor of HMH. Then, they evaluated the performances of the electrodialysis (ED) process to remove these 15 volatile compounds as well as trimethylamine, dimethylamine, and trimethylamine oxide, by testing the impact of both hydrolysate pH (4 and 7) and pulsed electric field (PEF) current modes (duration of a DC pulse and pause). The ED performance was compared with that of a deaerator by assessing three hydrolysate pH values (4, 7, and 10). The initial pH of HMH had a huge impact on the targeted compounds, while ED had no effect. Finally, the authors showed that the fouling formation, resulting from electrostatic and hydrophobic interactions between HMH constituents and ion-exchange membranes (IEM), the occurrence of water splitting at IEM interfaces, due to the reaching of the limiting current density, and the presence of water dissociation catalyzers were considered as the major limiting ED-process conditions.

Skolotneva et al. [5] focused on the electrochemical methods utilizing reactive electrochemical membranes (REMs) as a promising technology for efficient degradation and mineralization of organic compounds in natural, industrial, and municipal wastewaters. They proposed a two-dimensional (2D) model considering the transport and reaction of organic species with hydroxyl radicals generated at a TiO_x_ REM operated in flow-through mode, which takes into account convection, diffusion, and chemical reaction constants. This model allows the determination of unknown parameters of the system by treatment of experimental data and predicts the behavior of the electrolysis setup. The authors obtained a good agreement in the calculated and experimental degradation rate of a model pollutant (foulants molecules) at different permeate fluxes and current densities. The model also provides an understanding of the current density distribution over an electrically heterogeneous surface and its effect on the distribution profile of hydroxyl radicals and diluted species. Skolotneva et al. proved that the removal percentage of paracetamol increases with decreasing the pore radius by foulant molecules and/or increasing the porosity of the membrane. This effect becomes more pronounced as the current density increases. The model highlights how convection, diffusion, and reaction limitations have to be taken into consideration for understanding the effectiveness of the process.

Andreeva et al. [6] investigated scaling on the surface of a heterogeneous MK-40 cation-exchange membrane and on the same membrane, the surface layer of which contained polyaniline. The studies were carried out using a solution of a mixture of Na_2_CO_3_, KCl, CaCl_2_, and MgCl_2_ salts. Voltammetry and chronopotentiometry with the simultaneous control of the desalted solution pH provided information on the effect of current density on the concentration polarization of the membrane systems under study. A mineral scale on the surfaces of the studied membranes was found by scanning electron microscopy. Two limiting currents, which were observed on the current–voltage curve of the modified membrane, indicated the formation of a bipolar interface between the polyaniline layer with positively charged amino groups and the pristine membrane with negatively charged sulfone fixed groups. Enhanced water splitting due to the formation of this bipolar interface caused more significant scaling on the modified membrane compared to the commercial MK-40.

Nichka et al. [7] compared the effect of five different solution flow rates, corresponding to Reynolds numbers of 162, 242, 323, 404, and 485, combined with the use of pulsed electric field current mode to avoid protein fouling of the bipolar membrane (BPM) during electrodialysis of skim milk. They proved that application of PEF almost completely prevented fouling formation by proteins on the cationic interface of the BPM, regardless of the flow rate or Reynolds number. Indeed, under the PEF mode of current, the weight of protein fouling was negligible in comparison with the continuous current (CC) mode (0.07 ± 0.08 mg/cm^2^ vs. 5.56 ± 2.40 mg/cm^2^). When CC mode was applied, a Reynolds number equal to or higher than 323 corresponded to a minimal value of protein fouling of the BPM. The authors explain that the positive effect of both increasing the flow rate and using a PEF is due to the fact that during pauses, the solution flow flushes the accumulated protein from the membrane while at the same time there is a decrease in concentration polarization (CP) and consequently a decrease in H^+^ flux at the feed solution/cationic interface of the BPM, minimizing fouling formation and accumulation.

Gil et al. [8] focused on modifying the surface of the MK-40 cation-exchange membrane to reduce fouling in wastewater ED by enhancing electroconvection. The surface of this membrane was covered with the perfluorosulfonic acid (PFSA) polymer film doped with TiO_2_ nanoparticles. It was found that changes in surface characteristics conditioned by such modification lead to an increase in the limiting current density due to the stimulation of electroconvection, which develops according to the mechanism of electroosmosis of the first kind. The greatest increase in the current compared to the pristine membrane was obtained by modification with the film being 20 μm thick and containing 3 wt% of TiO_2_. The sample containing 6 wt% of TiO_2_ provided higher mass transfer in over-limiting current modes due to the development of nonequilibrium electroconvection. A 1.5-fold increase in the thickness of the modifying film reduced the positive effect of introducing TiO_2_ nanoparticles due to (1) partial shielding of the nanoparticles on the surface of the modified membrane and (2) a decrease in the tangential component of the electric force, which affects the development of electroconvection.

Bdiri et al. [9] used enzymatic agents as biological solutions for cleaning ion-exchange membranes fouled by organic compounds during ED treatments in the food industry. They tested the cleaning efficiency of three enzyme classes (β-glucanase, protease, and polyphenol oxidase) chosen for their specific actions on polysaccharides, proteins, and phenolic compounds, respectively, fouled on a homogeneous cation-exchange membrane (referred to as CMX-Sb) and used for tartaric stabilization of red wine by ED in industry. First, enzymatic cleaning tests were performed using each enzyme solution separately with two different concentrations (0.1 and 1.0 g/L) at different incubation temperatures (30, 35, 40, 45, and 50 °C). The evolution of membrane parameters, such as electrical conductivity, ion-exchange capacity, and contact angle was determined to estimate the efficiency of the membrane′s principal action as well as its side activities. Based on these tests, these authors determined the optimal operating conditions for optimal recovery of the studied characteristics. Then, they tested two strategies combining these enzymes. The first one consisted of cleaning the fouled membranes with three successive enzyme solutions. The second one combined the use of two enzymes simultaneously in an enzyme mixture. In each case they took into account the optimal conditions of the enzymatic activity (concentration, temperatures, and pH). This study led to significant results, indicating effective external and internal cleaning by the studied enzymes. For example, they obtained a recovery rate of at least 25% of the electrical conductivity, 14% of the ion-exchange capacity, and 12% of the contact angle, and demonstrated the presence of possible enzyme combinations for the enhancement of the global cleaning efficiency or reducing cleaning durations. These important results prove, for the first time, the applicability of enzymatic cleanings to membranes and the inertia of their action towards the polymer matrix to the extent that the choice of enzymes is specific to the fouling substrates.

## References

[1] Dammak L., Fouilloux J., Bdiri M., Larchet C., Renard E., Baklouti L., Sarapulova V., Kozmai A., Pismenskaya N. (2021). A Review on Ion-Exchange Membrane Fouling during the Electrodialysis Process in the Food Industry, Part 1: Types, Effects, Characterization Methods, Fouling Mechanisms and Interactions. Membranes.

[2] Pismenskaya N., Bdiri M., Sarapulova V., Kozmai A., Fouilloux J., Baklouti L., Larchet C., Renard E., Dammak L. (2021). A Review on Ion-Exchange Membranes Fouling during Electrodialysis Process in Food Industry, Part 2: Influence on Transport Properties and Electrochemical Characteristics, Cleaning and Its Consequences. Membranes.

[3] Kozmai A., Sarapulova V., Sharafan M., Melkonian K., Rusinova T., Kozmai Y., Pismenskaya N., Dammak L., Nikonenko V. (2021). Electrochemical Impedance Spectroscopy of Anion-Exchange Membrane AMX-Sb Fouled by Red Wine Components. Membranes.

[4] Todeschini S., Perreault V., Goulet C., Bouchard M., Dubé P., Boutin Y., Bazinet L. (2020). Assessment of the Performance of Electrodialysis in the Removal of the Most Potent Odor-Active Compounds of Herring Milt Hydrolysate: Focus on Ion-Exchange Membrane Fouling and Water Dissociation as Limiting Process Conditions. Membranes.

[5] Skolotneva E., Trellu C., Cretin M., Mareev S. (2020). A 2D Convection-Diffusion Model of Anodic Oxidation of Organic Compounds Mediated by Hydroxyl Radicals Using Porous Reactive Electrochemical Membrane. Membranes.

[6] Andreeva M.A., Loza N.V., Pis’menskaya N.D., Dammak L., Larchet C. (2020). Influence of Surface Modification of MK-40 Membrane with Polyaniline on Scale Formation under Electrodialysis. Membranes.

[7] Nichka V.S., Geoffroy T.R., Nikonenko V., Bazinet L. (2020). Impacts of Flow Rate and Pulsed Electric Field Current Mode on Protein Fouling Formation during Bipolar Membrane Electroacidification of Skim Milk. Membranes.

[8] Gil V., Porozhnyy M., Rybalkina O., Butylskii D., Pismenskaya N. (2020). The Development of Electroconvection at the Surface of a Heterogeneous Cation-Exchange Membrane Modified with Perfluorosulfonic Acid Polymer Film Containing Titanium Oxide. Membranes.

[9] Bdiri M., Bensghaier A., Chaabane L., Kozmai A., Baklouti L., Larchet C. (2019). Preliminary Study on Enzymatic-Based Cleaning of Cation-Exchange Membranes Used in Electrodialysis System in Red Wine Production. Membranes.

